# Blood Pressure Profile and N-Terminal-proBNP Dynamics in Response to Intravenous Methylprednisolone Pulse Therapy of Severe Graves’ Orbitopathy

**DOI:** 10.3390/ijms19102918

**Published:** 2018-09-26

**Authors:** Piotr Miskiewicz, Justyna Milczarek-Banach, Tomasz Bednarczuk, Grzegorz Opolski, Renata Glowczynska

**Affiliations:** 1 Department of Internal Medicine and Endocrinology, Medical University of Warsaw, Banacha 1a, 02097 Warsaw, Poland; piotr.miskiewicz@wum.edu.pl (P.M.); justynamilczarekbanach@gmail.com (J.M.-B.); tbednarczuk@wum.edu.pl (T.B.); 2 First Department of Cardiology, Medical University of Warsaw, Banacha 1a, 02097 Warsaw, Poland; grzegorz.opolski@wum.edu.pl

**Keywords:** glucocorticoids, methylprednisolone, ambulatory blood pressure monitoring, blood pressure, hypertension, graves’ orbitopathy, NT-proBNP, Graves’ disease, pulse therapy

## Abstract

Hypercortisolemia is associated with increased risk of hypertension. Natural and synthetic glucocorticoids (GCs) have different effects on blood pressure (BP). The effect of synthetic GCs on BP depends on the dose, treatment duration, type of GCs, and route of administration. Intravenous methylprednisolone (IVMP) pulse therapy is the first line of treatment for severe Graves’ orbitopathy (GO). The aim of this study was to evaluate influence of IVMP pulses on BP and N-terminal pro-brain natriuretic peptide (NT-proBNP) dynamics. A total of 32 patients with GO were treated with one IVMP pulse every week for 12 weeks. We performed 48-h BP monitoring (24-h before and 24-h after IVMP) and measured NT-proBNP before, 24 h, and 48 h after the 1st, 6th, and 12th IVMP pulse. Mean BP did not change after any of the pulses. We did not observe an increase in maximal systolic BP or mean nocturnal BP, except after the last pulse. Additionally, the dipping phenomenon was less frequent after the last pulse. We found a significant increase in median NT-proBNP levels after all analyzed pulses. Our study suggests that IVMP may have an unfavorable cumulative effect on BP. Variation in NT-proBNP concentration indicates a compensatory effect of brain natriuretic peptide secretion.

## 1. Introduction

High dose intravenous (i.v.) glucocorticoid (GC) pulse therapy is an effective immunosuppressive treatment used in various inflammatory and autoimmune diseases [1]. One of the indications for this treatment is Graves’ orbitopathy (GO). High-dose GCs, preferably via the i.v. route, are the first line of treatment for active, moderate-to-severe GO. The optimal cumulative dose appears to be 4.5–5.0 g of intravenous methylprednisolone (IVMP) [2]. According to the European Group On Graves’ Orbitopathy (EUGOGO) recommendations, this therapy was proven to be more effective compared to high-dose oral GC therapy and is associated with a lower recurrence rate [2]. EUGOGO recommends treatment with IVMP for patients with moderate-to-severe and very severe GO (dysthyroid optic neuropathy−DON). For patients with active, moderate-to-severe GO, EUGOGO advices 12 pulses of IVMP with a treatment duration of 12 weeks with single weekly i.v. pulses. The first six weeks is 0.5 g IVMP, the next six weeks is 0.25 g IVMP. In some patients without improvement after the first cycle of IVMP, an additional cycle with a cumulative dose of 4.5 g of methylprednisolone is prescribed using the same treatment scheme. In patients with DON, which is associated with the risk of vision loss, the treatment schedule is more aggressive (1.0 g in three consecutive days repeated after two weeks in case of lack of improvement) [2].

However, a major concern was raised given reports of fatal side effects that may be associated with this therapy [1,3]. In a meta-analysis performed by Zang et al., calculated morbidity and mortality rates in patients with GO treated with i.v. GCs were 6.5% and 0.6%, respectively [4]. Therapy with i.v. GCs may be associated with mild, moderate, and severe cardiovascular adverse events including cardiac arrhythmias, pulmonary embolism, myocardial infarction, exacerbation of heart failure, and increased blood pressure (BP) [1,3,5,6]. 

Synthetic GCs on BP appear to have a lesser impact than endogenous hypercortisolemia. Synthetic GCs with minimal mineralocorticoid activity stimulate the increase in BP without any urinary sodium retention, increase in body weight, or plasma volume [7]. However, very high doses overwhelm the protective role of 11β-hydroxysteroid dehydrogenase type 2 (11β-HSD2) and can influence the mineralocorticoid receptor. Other potential mechanisms of hypertension induction in hypercortisolemia include increased pressor responsiveness to angiotensin II [8], influence on the sympathetic nervous system [9], and the vasoregulator system. 

A consensus on the monitoring of patients during and after IVMP pulse administration has not yet been established [2,10]. The effects of IVMP treatment on hemodynamic markers have not yet studied in detail. It is of great importance to evaluate and prevent the risk of severe and fatal side effects of IVMP treatment. Thus, we decided to monitor BP before, during, and after GC pulse administration and assess acute changes in N-terminal pro-brain natriuretic peptide (NT-proBNP) as a hemodynamic marker. 

## 2. Results

### 2.1. Ambulatory Blood Pressure Monitoring (48-h)

**Figure 1:**
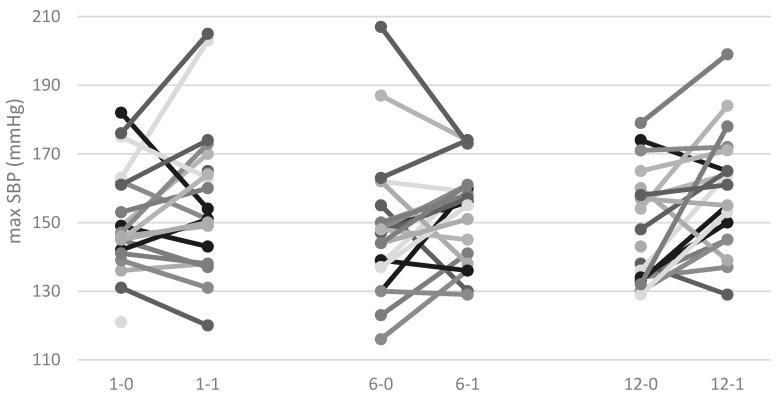
Changes in maximal systolic blood pressure. max SBP is maximal systolic blood pressure (BP), 1-0 is 24-hours (h) before first intravenous methylprednisolone (IVMP) infusion, 1-1 is 24-h after the beginning of first IVMP infusion, 6-0 is 24-h before sixth IVMP infusion, 6-1 is 24-h after the beginning of sixth IVMP infusion, 12-0 is 24-h before 12th IVMP infusion, and 12-1 is 24-h after the beginning of 12th IVMP infusion. The different colors of lines “grey, white, black…” define BP measurements in examined patients.

**Figure 2:**
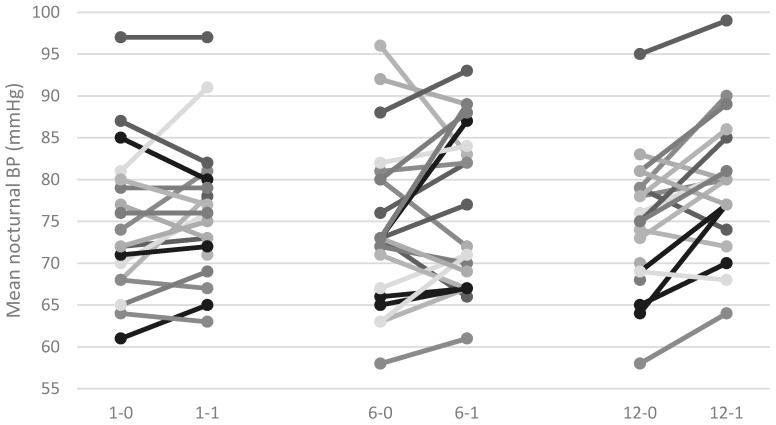
Changes in mean nocturnal blood pressure. BP is blood pressure, 1-0 is 24-hours (h) before first intravenous methylprednisolone (IVMP) infusion, 1-1 is 24-h after the beginning of first IVMP infusion, 6-0 is 24-h before sixth IVMP infusion, 6-1 is 24-h after the beginning of sixth IVMP infusion, 12-0 is 24-h before 12th IVMP infusion, and 12-1 is 24-h after the beginning of 12th IVMP infusion. The different colors of lines “grey, white, black…” define BP measurements in examined patients.

Results from 48-h ambulatory blood pressure monitoring (ABPM) were available from 22 patients. Table 1 shows changes in mean BP, mean nocturnal BP, maximal systolic blood pressure (SBP) between day before and day after IVMP pulse during the 1st, 6th, and 12th pulse. In Figure 1 and Figure 2, we present the changes in mean nocturnal BP and maximal SBP, respectively. There were no statistically significant changes in the mean BP during the entire therapy or in the maximal SBP and mean nocturnal BP after the 1st and 6th pulse. However, we noticed during the last, 12th, pulse a significant increase in maximal SBP and mean nocturnal BP, as shown in Figure 1 and Figure 2 and Table 1. Additionally, we observed that the dipping phenomenon was statistically less frequent 24-h after the last IVMP pulse in comparison to 24-h after the first IVMP pulse (Table 2).

### 2.2. Neurohormonal Biomarker NT-proBNP

**Figure 3:**
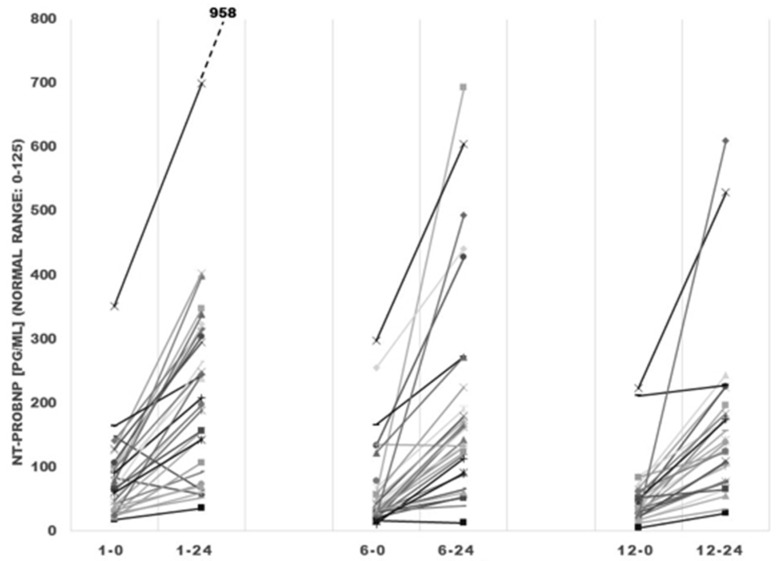
Changes in NT-proBNP values in the selected points of the study. NT-proBNP—N-terminal pro-brain natriuretic peptide, 1-0: before first intravenous methylprednisolone (IVMP) infusion, 1-24: 24 hours (h) after first IVMP infusion, 6-0: before 6th IVMP infusion, 6-24: 24 h after sixth IVMP infusion, 12-0: before 12th IVMP infusion, 12-24: 24 h after 12th IVMP infusion. The different colors of lines “grey, white, black…” define changes in NT-proBNP values in examined patients.

The baseline median value of NT-proBNP before the treatment was 70 (39–98) pg/mL (normal range: <125 pg/mL). There was a statistically significant increase in median NT-proBNP levels between before, and 24 and 48 h after IVMP pulse in all of the analyzed pulses (1st, 6th, and 12th) (Table 3 and Figure 3). The highest increase in the NT-proBNP was observed 24 h after the administration of IVMP in each pulse. We observed a significant decrease in median NT-proBNP levels between the beginnings of the 1st and 6th IVMP pulses (*p* = 0.024), as well as the 1st and 12th IVMP pulses (*p* = 0.0016).

### 2.3. Additional Biomarker of Cardiomyocyte Injury—Troponin I (TnI) 

The basic values of TnI were in the normal range in all patients. Only one of the participants reached normal TnI cutoff level (0.056 ng/mL) during any of the analyzed pulses. The increase in TnI level to 0.07 ng/mL was observed in one woman 48 h after the 12th IVMP pulse, but without any clinical symptoms of coronary heart disease. There were no statistically significant changes in TnI levels across the analyzed IVMP pulses.

### 2.4. Echocardiography 

Patients underwent echocardiographic examination before the 1st and after the 12th pulse (measurements were available in 22 participants). Median values of ejection fraction (EF) remained almost unaffected by the GC therapy: 65% before treatment (range: 56–71) vs. 65% after treatment (range: 55–73). The increase in EF was observed in 36% (*n* = 8) patients (mean value of 3%), a decrease in 41% (*n* = 9) patients (mean value of 3%), and EF in 23% (*n* = 5) patients remained unchanged. 

### 2.5. Cardiovascular Events

We did not observe any serious adverse cardiovascular events.

## 3. Discussion

Hypercortisolemia is associated with increased risk of hypertension. Natural and synthetic GCs have different effects on BP [11]. Hypertension is diagnosed in about 80% of patients with endogenous hypercortisolemia [12]. The impact of synthetic GCs on BP seems to be lesser especially during the first months of exposure [11]. The impact depends on the dose, treatment duration, type of GCs, and route of administration. The difference occurs probably between patients treated with daily oral GCs and intravenous pulse therapy; this has not yet been fully investigated. The mechanisms involved in the development of hypertension in patients with GC excess are complex and only partially understood. Synthetic GCs have minimal mineralocorticoid activity, and produce elevations in BP without any urinary sodium retention, increase in body weight, or plasma volume [7]. Renal mineralocorticoid receptor activation occurs in extremely elevated circulating cortisol levels that overwhelm the protective role of 11 β-HSD2. Possible mechanisms of hypertension development have been postulated, such as increased pressor responsiveness to angiotensin II [8] or influence on the sympathetic nervous system [9]. The influence of GCs on the vasoregulator system probably plays a crucial role in hypertension development. Many substances with vasoregulatory properties have been reported to contribute to hypertension secondary to GCs excess. Increased levels of endothelin-1 and erythropoietin have been suggested as possible factors of GC-induced hypertension [9]. Most studies on vasodilators have demonstrated effects of GC excess on the nitric oxide pathway through different mechanisms; one of them is inhibition of nitric oxide synthase (NOS) expression [9].

In the presented study, we analyzed the influence of IVMP therapy on the circadian BP pattern, biochemical markers of heart failure (NT-proBNP), and myocardial injury (TnI), as well as the echocardiographic parameter (EF). We did not observe any changes in the 24-h mean BP in all three evaluation periods (after first, sixth, and 12th pulse of IVMP) or in mean nocturnal BP and maximal SBP after first and sixth pulse of IVMP. However, after the last (12th) IVMP pulse, we observed an increase in mean nocturnal BP, maximal SBP, and a trend in a non-dipping BP profile. 

This is the first study suggesting the potential cumulative effect of IVMP administration on the circadian BP profile. The fast reaction of BP to IVMP pulses was analysed in a retrospective study by Yong et al. (1.0 g of IVMP) [10] and a prospective study by Lauridsen et al. (0.5 g of IVMP) [13]. There was an observed decrease in SBP and DBP at 30 and 60 min after an IVMP pulse in the first study and no change in SBP with a reduction in DBP 90 min after an IVMP pulse in the second study. In a prospective study, Brootman et al. noticed an opposite reaction of BP after five days’ treatment with 3 mg of dexamethasone twice daily [14]. In the intervention group, SBP increased compared to baseline evaluation; however, in the placebo group, DBP did not change. This study showed that dexamethasone, with negligible mineralocorticoid activity, suppresses endogenous cortisol and impacts the reduction in aldosterone. The lack of BP increases of in the studies of Yong et al. and Lauridsen et al., and the increase in SBP in the study by Brootman et al. indicate the difference between the fast reaction of intravenous GCs and the cumulative reaction of oral GCs on BP. Finally, the non-dipping BP profile found at the end of our IVMP course was also found in patients with hypercortisolemia in a study by Imai et al. [15]. Loss of nocturnal BP promotes cardiac, renal, and vascular injury.

Simultaneously, we observed a statistically significant increase in median NT-proBNP in all of the analyzed pulses, the greatest occurring 24 h after the administration of IVMP. The median increase in NT-proBNP levels during first 24 h was less pronounced after the 12th pulse. We observed a decrease in median NT-proBNP between basal values, before administration of the 1st, 6th, and 12th IVMP pulses. The increase in NT-proBNP after IVPM pulses indicated the elevated release of brain natriuretic peptide (BNP) that is secreted by cardiomyocytes in response to stretch [16]. BNP may play a crucial role in maintaining cardiovascular hemodynamics with an impact on BP regulation. BNP may reduce BP through several mechanisms, such as increased renal perfusion and natriuresis, renin-angiotensin-aldosterone system inhibition, and arterial dilation [17]. The induction of the *BNP* gene results in the production and secretion of prohormone proBNP_1-108_. This is cleaved into the biologically active BNP_1-32_ and biologically inert and more stable NT-proBNP [18]. The same reaction of BNP was observed by Brootman et al. [14]. Lack of mineralocorticoid activity of dexamethasone indicates that increased BNP is probably not associated with expansion of plasma volume [14]. Lauridsen et al. observed after an IVMP pulse (0.5 g) a simultaneous increase in plasma atrium natriuretic peptide (ANP), plasma arginine vasopressin, and urinary excretion of aquaporin-2 without changes of urinary excretion of sodium and free water clearance [13]. The authors suggested that increased tubular absorption of water and sodium is antagonized by compensatory increase of ANP. Direct stimulation of dexamethasone on plasma ANP concentration within two hours and ANP mRNA within 30 min was documented in a study performed by Dananberg et al. [19]. Finally, NT-proBNP increases in patients with endogenous, long lasting hypercortisolemia. The concentration of NT-proBNP is higher in patients with overt Cushing’s disease than in those with subclinical Cushing’s syndrome and non-functioning adenoma [20].

We did not analyze the influence of IVMP in subgroups of patients (e.g., male vs. female) as the study group was too small to perform such analysis. However, it would be interesting to include such analysis in a larger group of patients according to sex, age, and comorbidities. 

According to previous studies and our results, we consider therapy with IVMP pulses administered weekly as safe according to BP changes in normotensive patients and patients with well-controlled BP before treatment. However, during the cycle of treatment, an unfavorable cumulative effect on BP was observed after the last pulse. The study indicates a compensatory increase in BNP after every pulse of IVMP. We hypothesize that this effect could be reduced when the therapy lasts longer. This could explain why we observed the increase in maximal SBP and mean nocturnal BP and higher prevalence of non-dipping BP profile with lower median increase in NT-proBNP in the last evaluated pulse of IVMP. This is the first study concerning fast and cumulative influence of IVMP pulses on circadian BP.

We did not observe any changes in echocardiography before and after treatment. We found no clinical symptoms of heart failure or acute exacerbation of heart failure in our patients. 

## 4. Materials and Methods 

### 4.1. Study Population

This study was a prospective, open, non-randomized, clinical study involving consecutive patients with active, moderate-to-severe GO treated with IVMP pulse therapy according to European Group On Graves’ Orbitopathy (EUGOGO) recommendations [2]. Patients were admitted to the Department of Endocrinology and Internal Medicine, Medical University of Warsaw in the period between 2011 and 2015. The inclusion criteria were as follows: (1) active, moderate-to-severe GO according to EUGOGO classification; (2) age ≥18 years; (3) euthyroidism (patients with hyperthyroidism treated with anti-thyroid drugs, after radiotherapy/surgical treatment on levothyroxine (L-T4) therapy if necessary, with euthyroid Graves’ disease, or with Hashimoto disease on L-T4 therapy); and (4) completion of at least first six IVMP pulses. Exclusion criteria were as follows: (1) cardiovascular morbidity, such as chronic heart failure and/or coronary heart disease; (2) uncontrolled hypertension defined as SBP more than 140 mmHg and/or DBP more than 90 mmHg; (3) contraindications to IVMP therapy; and (4) previous GCs treatment in the last 6 months. Informed consent was obtained from each patient. There were 32 patients included into the study; all but 4 underwent the entire treatment schedule. Four persons received the first six pulses. Detailed patient characteristics are provided in Table 4. 

### 4.2. Study Design 

**Figure 4:**
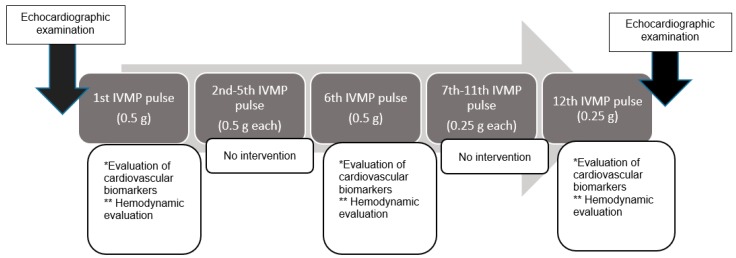
The scheme of study design. * Evaluation of cardiovascular biomarkers included examination of N-terminal pro-brain natriuretic peptide, troponin I, 24 and 48 h after intravenous methylprednisolone (IVMP) pulse. ** Hemodynamic evaluation included 48-h ambulatory blood pressure monitoring during 24-h before and 24-h after IVMP infusion.

After enrolment, all participants were qualified for treatment with 4.5 g IVMP pulses (cumulative dose of methylprednisolone (MP)), treatment duration 12 weeks in once-weekly intravenous pulses, with each pulse in the first 6 weeks 0.5 g MP, and next 6 weeks 0.25 g MP) (Figure 4).

Laboratory tests, such as NT-proBNP and TnI, were performed before, and 24 and 48 h after IVMP infusion during the following pulses: 1st, 6th, and 12th. Patients underwent echocardiographic examination before the 1st and after the 12th pulse. The 48-h ABPM (for 24-h before and 24-h after IVMP infusion) was recorded during the following pulses: 1st, 6th, and 12th. Standard monitoring and prophylaxis were performed before every pulse: evaluation of clinical status, symptoms of infection, BP monitoring, glucose level monitoring, monitoring of liver enzymes, and prophylaxis of osteoporosis. The primary endpoint was the change in BP and laboratory-documented change in cardiovascular biomarkers, which indicated the influence of IVMP on the cardiovascular system. The secondary endpoints included adverse cardiovascular events (death, myocardial infarction, stroke, severe heart failure, hypertensive crisis, and cardiac arrhythmias). The study protocol conformed to the ethical guidelines of the 1975 Declaration of Helsinki as reflected in a priori approval by the institution’s human research committee (KB 42/2011; 15 March 2011). The study was registered on clinicaltrials.gov with the number NCT03590080.

### 4.3. Ambulatory BP Monitoring (48-h)

We performed 48-h BP monitoring (24-h before and 24-h after the 1st, 6th, and 12th IVMP pulses). All measurements of ambulatory BP were recorded by devices certified by the British Hypertension Society. ABPM recorders were programmed to register one BP measurement every 20 min during the awake period and every 30 min during the sleeping period. Bedtime and wake-up time were set to each patient according to their individual sleeping and waking habits. The parameters evaluated by ABPM and analyzed in the study were: mean BP from 24-h of BP monitoring and calculated as follows: (SBP-DBP) × 1/3 + DBP, mean nocturnal BP (mean BP from the sleeping period calculated with the formula mentioned above), and maximal SBP (the maximal single result from SBP measurement from 24-h of BP monitoring). The measurements, techniques, and device settings were in agreement with the current guidelines for hypertension diagnosis and management [21]. The appropriate cuff size was provided individually for every patient. 

The absence of BP dipping phenomenon during sleeping was defined as a reduction in nocturnal SBP equal to or less than 10% in relation to the SBP when awake according to ABPM readings. Thus, we categorized individuals as dippers when the sleep-time-relative SBP decline was ≥10% and non-dippers when SBP decline was <10%.

### 4.4. NT-proBNP Measurements

All assays were performed in sera obtained from venous blood samples and analyzed immediately after blood sampling in The Central Laboratory in our hospital. NT-proBNP was measured using a Flex^®^ Reagent Cartridge and Dimension^®^ EXLTM integrated chemistry system (Newark, NJ, USA) with a LOCI^®^ Module (Siemens HealthCare Diagnostics Ltd., Camberley, UK). The cutoff value of NT-proBNP typical for hemodynamic stress in the left ventricle was defined as ≥125 pg/mL [22]. 

### 4.5. Additional Laboratory Measurements

In addition to NT-proBNP, TnI was measured. Analysis of TnI was performed on Siemens Dimension System (ExL LOCI TnI assay, Newark, NJ, USA). TnI values ≥0.056 ng/mL above the 99th percentile were reported as positive. 

### 4.6. Echocardiography

All echocardiographic examinations were performed using the Philips Medical Systems (Andover, MA, USA) with a sector 5-1-MHz. Measurements were performed according to the American Society of Echocardiography chamber quantification guidelines [23].

The two-dimensional echocardiography studies were performed and interpreted by a board-certified cardiologist certified in transthoracic echocardiography. The parameter examined and analyzed in this study was the ejection fraction, which was calculated from end diastolic volume (EDV) and end systolic volume (ESV) estimates, using the following formula: EF = (EDV − ESV)/EDV.

### 4.7. Statistical Analysis 

Continuous variables are expressed as median values (lower quartile (Q1)—upper quartile (Q3). Categorical variables are presented as numbers (N) or percentage values (%). Comparisons between continuous variables (NT-proBNP, TnI, mean BP, mean nocturnal BP, and maximal SBP) were assessed before and after selected IVMP pulses using a non-parametric Wilcoxson matched Pairs test. Analysis of paired nominal data, such as presence of BP dipping during sleep before and after IVMP pulses, was performed using McNemar’s test. Results with *p* value < 0.05 were considered statistically significant. All statistical analyses were made using STATISTICA ver.12.0 (Statsoft, Kraków, Poland).

## 5. Conclusions

Our study suggests that treatment with intravenous methylprednisolone once every week may have an unfavorable cumulative effect on BP. Variation in NT-proBNP concentration after IVMP indicates the compensatory effect of brain natriuretic peptide secretion.

## Figures and Tables

**Table 1:** Changes in 24-h blood pressure during selected intravenous methylprednisolone (IVMP) pulses in the entire group of patients (N = 22).

IVMP Pulse	Before Pulse Median (Q1–Q3, mmHg)	After Pulse Median (Q1–Q3, mmHg)	*p* (Before Pulse vs. After Pulse)
Mean BP			
1st	84 (75–88)	83 (77–87)	0.64
6th	81 (77–85)	83 (75–87)	0.40
12th	82 (78–85)	83 (79–88)	0.23
Mean nocturnal BP			
1st	73 (68–81)	76 (72–80)	0.39
6th	73 (67–80)	72 (67–84)	0.24
12th	75 (69–79)	80 (74–85)	0.005
Maximal SBP			
1st	147 (142–162)	154 (143–165)	0.37
6th	148 (138–155)	157 (138–161)	0.21
12th	146 (134–159)	161 (145–171)	0.01

BP: blood pressure, SBP: systolic blood pressure, IVMP: intravenous methylprednisolone, N: number of patients, Q1: lower quartile, Q3: upper quartile, before pulse: 24-hours (h) BP evaluation before infusion of IVMP, after pulse: 24-h BP evaluation after infusion of IVMP.

**Table 2:** Occurrence rate of dipping phenomenon during selected intravenous methylprednisolone pulses (N = 20).

IVMP Pulse	Before Pulse N (%)	After Pulse N (%)	*p* (Before Pulse vs. After Pulse)
1st	10 (50)	7 (35)	0.77
6th	9 (45)	8 (40)	0.58
12th	13 (65)	2 (10)	0.45
	*p* (1st vs. 6th) 1.00, *p* (1st vs. 12th) 0.50	*p* (1st vs. 6th) 0.37, *p* (1st vs. 12th) 0.02	

IVMP: intravenous methylprednisolone, N: number of patients, before pulse: 24-hours (h) blood pressure (BP) evaluation before infusion of IVMP, after pulse: 24-h BP evaluation after infusion of IVMP. Dipping phenomenon during sleeping was defined as a reduction of nocturnal systolic BP (SBP) more than 10% in relation to the SBP during awake period.

**Table 3:** Changes in N-terminal pro-brain natriuretic peptide levels during selected IVMP pulses (N = 32).

IVMP Pulse	Before Pulse Median (Q1–Q3), pg/mL	After 24 h Median (Q1–Q3), pg/mL	After 48 h Median (Q1–Q3), pg/mL	*p* (Before Pulse vs. After 24 h)	*p* (Before Pulse vs. After 48 h)
1st	70 (39–98)	203 (100–310)	187 (111–235)	0.000003	0.000005
6th	32 (24–62)	150 (91–225)	141 (98–212)	0.000002	0.000011
12th	38 (23–60)	134 (78–190)	96 (64–216)	0.000004	0.000012

IVMP: intravenous methylprednisolone, Q1: lower quartile, Q3: upper quartile, N: number of patients.

**Table 4:** Baseline characteristics of study population (N = 32).

Male, % (N)	38 (12)
Age, years	52 ± 11
**Etiology of GO**	
GD, % (N)	78 (25)
AITD, % (N)	22 (7)
**Smoking**	
Current, % (N)	53 (17)
Former, %(N)	28 (9)
Never, % (N)	19 (6)
CAS (range 1–7)	4 ± 2
BMI (kg/m^2^)	26.0 ± 4.1
**Comorbidities**	
Hypertension, % (N)	41 (13)
diabetes % (N)	0 (0)
CHD, % (N)	0 (0)
**Medications**	
β-blockers, % (N)	41 (13)
ACEI, % (N)	25 (8)
ARB, % (N)	6 (2)
Ca-blockers, % (N)	19 (6)
Statins, % (N)	16 (5)
Diuretics, % (N)	9 (3)
L-thyroxine, % (N)	66 (21)
Thyrostatics, % (N)	44 (14)
**Laboratory measurements**	
TSH (range: 0.27–4.2 µIU/mL)	1.6 ± 1.6
fT4 (range: 12–22 pmol/L)	16.8 ± 3.5
TBII (N < 1.73 IU/L)	9.8 ± 11.6

ACEI: Angiotensin Converting Enzyme inhibitors, AITD: autoimmune thyroid disease other than Graves’ disease, ARB: Angiotensin Receptor Blockers, ASA: acetylsalicylic acid, BMI: body mass index, CAS: Clinical Activity Score, CHD: coronary heart disease, fT4: free thyroxine, GD: Graves’ disease, GO: Graves’ orbitopathy, N: number, TBII: thyrotropin binding inhibitory immunoglobulins, TSH: thyrotropin. Laboratory tests (TSH, FT4, TBII) are presented as a mean value ± standard deviation.

## Abbreviations

ABPM	ambulatory blood pressure monitoring
BMI	body mass index
BP	blood pressure
BNP	brain natriuretic peptide
DBP	diastolic blood pressure
EF	ejection fraction
EUGOGO	European Group On Graves’ Orbitopathy
GCs	glucocorticoids
GO	Graves’ orbitopathy
IVMP	intravenous methylprednisolone
MP	methylprednisolone
NT-proBNP	N-terminal pro-brain natriuretic peptide
SBP	systolic blood pressure
TnI	troponin I
